# FtMYB16 interacts with Ftimportin‐α1 to regulate rutin biosynthesis in tartary buckwheat

**DOI:** 10.1111/pbi.13121

**Published:** 2019-05-01

**Authors:** Jinbo Li, Kaixuan Zhang, Yu Meng, Qiong Li, Mengqi Ding, Meiliang Zhou

**Affiliations:** ^1^ Institute of Crop Sciences Chinese Academy of Agricultural Sciences Beijing China; ^2^ Life Science College Luoyang Normal University Luoyang China; ^3^ College of Landscape and Travel Agricultural University of Hebei Baoding China; ^4^ School of Nursing Hunan University of Chinese Medicine Changsha China

**Keywords:** importin, MATE transporter, MYB transcription factor, nucleo‐cytoplasmic, trafficking, rutin, buckwheat

Tartary buckwheat (*Fagopyrum tataricum*) is well known to enrich in rutin among other buckwheat species (Figure 1a; Sytar *et al*., 2014). The subgroup 4 of R2R3‐MYB transcription factors (TFs), FtMYB11/13/14/15/16, are key repressors of rutin biosynthesis in buckwheat (Matsui *et al*., 2004; Zhang *et al*., 2018; Zhou *et al*., 2017). However, FtMYB16 is lack of the GY/FDFLGL motif (SID motif) and specially acts as a repressor on rutin biosynthesis in root (Zhang *et al*., 2018; Zhou *et al*., 2015). The SID motif contributes to the protein interaction with an importin‐β‐like protein that mediates the MYB nuclear trafficking (Zhao *et al*., 2007; Zhou *et al*., 2017). Here, we show that FtMYB16 interacts with Ftimportin‐α1 (FtPinG0006805200) to directly mediate the rutin biosynthesis *via* investigation of the rutin concentration and the kinetics on biomass growth of *F. tataricum* hairy roots overexpressing and silencing *FtMYB16*. It was found that the maximal rutin yield and biomass were obtained when the hairy roots were 20 days old. The hairy root culture of *F. esculentum* has characterized maximum biomass and high chlorogenic acid level on 20–21 days old as well (Gabr *et al*., 2012). The *FtMYB16* silencing lines grew faster and *FtMYB16* overexpressing lines grew slower than those of the empty vector (EV) lines, especially during 12–20 days. The rutin levels in *FtMYB16* overexpressing lines were lower compared to those of the *FtMYB16* silencing lines and EV (Figure 1b). These results indicated that FtMYB16 could repress both rutin accumulation and root growth.

**Figure 1:**
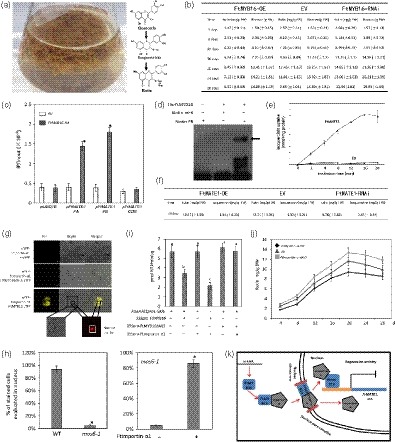
FtMYB16 regulates *FtMATE1* for rutin biosynthesis. (a) Rutin biosynthesis pathway and hairy root cultures of *F. tataricum*. (b) Rutin content and fresh weight in different genotypes of 4‐, 8‐, 12‐, 16‐, 20‐, 24‐ and 28‐day‐old *F. tataricum* hairy roots. Values are means ± SD of three biological repeats of each independent transgenic line. The transgenic hairy root lines generated from transformations with strains *A. rhizogenes* A4 harbouring T‐DNA contain no open reading frame (empty vector, EV; Lines 1, 3, 4), *FtMYB16*‐OE (Lines 1, 2, 4) and *FtMYB16*‐RNAi (Lines 1, 4, 6). (c) Direct binding of FtMYB16 with the promoter of *FtMATE1* by ChIP assays. ChIP assays were conducted by RT‐PCR after normalizing with the input DNA. The fragment of *FtMATE1* coding sequence and the reference gene *UBQ10* promoter were used as a negative control. Significant differences between values are indicated with asterisks as tested by Student's *t*‐test (*P* < 0.05). (d) EMSA of a probe or mutant probe *FtMATE1* promoter fragment B and its mutant with His‐*FtMYB16* purified from *Escherichia coli* BL21 (DE3). The arrow indicates the protein probe complex. (e) Time‐dependent uptake into vesicles from yeast cells transformed with *FtMATE1* or empty vector (EV) to 100 μm isoquercitrin. (f) Rutin and isoquercitrin contents in different genotypes of 20‐day‐old *F. tataricum* hairy roots. The transgenic hairy root lines generated from transformations with strains *A. rhizogenes* A4 harbouring T‐DNA contain no open reading frame (empty vector, EV; Lines 1, 2, 3), *FtMATE*1‐OE (Lines 3, 6, 9) and *FtMATE1*‐RNAi (Lines 2, 4, 5). Values are means ± SD of three biological repeats of each independent transgenic line. Letters indicate statistically significant differences compared with each other (*P* < 0.05, post hoc Tukey's HSD test). (g) FtMYB16 interacts with Ftimportin‐α1 in planta by BiFC assays. YFP fluorescence images alone or merged with bright‐field images of Arabidopsis cell suspension protoplasts co‐transfected with constructs encoding the indicated fusion proteins with YFP at the C‐terminus or the N‐terminus, RFP nuclear marker as a nuclear‐localized analysis. Scale bar = 10 μm. (h) Quantification of YFP‐FtMYB16 nuclear localization or with Ftimportin‐α1 in WT and *mos6‐1* mutant plant mesophyll protoplasts. At least 200 mesophyll protoplasts from each transformation were counted. Error bars indicate the ± SD from three independent biological replicates. Asterisks indicate statistically significant differences compared with each other (*P* < 0.05, Student's *t*‐test). (i) Arabidopsis cell suspension protoplasts were cotransformed with 2 μg reporter plasmid of *FtMATE1* pro1‐GUS and 2 μg of effector plasmids of 35S:: *FtMYB16*, 35S:: *FtMYB16* ΔNLS and 35S:: *Ftimportin‐*α*1*. Values represent means ± SE of triplicate experiments. Letters indicate statistically significant differences compared with each other (*P* < 0.05, post hoc Tukey's HSD test). (j) Rutin contents in different genotypes of 4‐, 8‐, 12‐, 16‐, 20‐, 24‐ and 28‐day‐old *F. tataricum* hairy roots. The transgenic hairy root lines generated from transformations with strains *A. rhizogenes* A4 harbouring T‐DNA contain no open reading frame (empty vector, EV; Lines 2, 4, 5), *Ftimportin‐*α*1*‐OE (Lines 1, 3, 6) and *Ftimportin‐*α*1*‐RNAi (Lines 1, 3, 9). Values are means ± SD of three biological repeats of each independent transgenic line. (k) A model of the role of Ftimportin‐α1 in the regulation of the FtMYB16 nucleo‐cytoplasmic trafficking in mediating rutin metabolism in Fagopyrum. The cytoplasmic cargo protein FtMYB16 binds to importin‐α Ftimportin‐α1; then, the complex FtMYB16/importin‐α translocates FtMYB16 into the nucleus, and this nucleo‐cytoplasmic trafficking signalling pathway depends on their protein interaction domain, such as NLS motif. FtMYB16 not only represses some key enzyme genes, such as *FtPAL*, in rutin biosynthesis, but also represses the gene expression of MATE transporter *FtMATE1* which transports isoquercitrin for rutin metabolism. Additionally, Ftimportin‐α1 functions as a corepressor on the transcriptional activity of FtMYB16 on its target genes.

Mining of *F. tataricum* transcriptome databases led to the identification of the multidrug and toxic compound extrusion (MATE) transporter gene *FtMATE1* (FtPinG0302610000) and the cell cycle regulator *Ftcyclin‐1* (FtPinG0003082800), which were tightly co‐expressed with *FtMYB16* and highly expressed in root (Logacheva *et al*., 2011). To assess whether FtMYB16 can directly regulate the *FtMATE1* gene expression, we performed chromatin immunoprecipitation (ChIP) analysis using 35S::*FtMYB16‐HA* overexpressing *F. tataricum* hairy root lines. As shown in Figure 1c, FtMYB16 was able to bind the fragment A (−782 to −774: AACTAGTTG) and B (−550 to −543: TTAAGTTG) of the *FtMATE1* promoter **(**Kelemen *et al*., 2007 **)**. Electrophoresis mobility shift assay (EMSA) further confirmed that FtMYB16 interacted with the fragment B (Figure 1d). Promoter analysis of the *Ftcyclin‐1* gene revealed that its promoter also contained the group I DNA motif. Taken together, FtMYB16 directly repressed the *FtMATE1* gene expression *via* one *cis*‐element of the *FtMATE1* promoter that belongs to the R2R3‐MYB binding group I DNA motif.

It has been reported that MATE transporters are essential for the vacuolar sequestration of flavonoid glucosides in plants (Marinova *et al*., 2007; Zhao *et al*., 2011). Here, the FtMATE1 shares 75% identity with AtTT12 from Arabidopsis. Microsomal vesicles assay showed that FtMATE1 mediated the uptake of glucosylated flavonoid isoquercitrin depending on the ATP (Figure 1e); however, it did not transport quercetin based on the studies of time course and concentration dependence. To confirm that FtMATE1 is a functional transporter involved in the rutin accumulation, we investigated the rutin concentration of *F. tataricum* hairy roots overexpressing and silencing *FtMATE1*. As shown in Figure 1f, the *FtMATE1* silencing lines showed lower rutin and higher isoquercitrin accumulation compared with other lines. These results indicated that isoquercitrin was transported by FtMATE1.

To identify proteins that interact with FtMYB16, yeast two‐hybrid (Y2H) screenings were performed using GAL4 DNA‐binding domain (BD)‐fused full‐length FtMYB16 as bait. Interestingly, an importin‐α protein named Ftimportin‐α1, homolog of Arabidopsis MOS6, was identified as the FtMYB16 interacting partner. Further Y2H tests demonstrated that FtMYB16 interacted with Ftimportin‐α1 depending on its NLS motif (KRRLLARRQSFTRK). Bimolecular fluorescence complementation (BiFC) assay observed strong YFP signals in the nucleus and cytoplasm of Arabidopsis cell suspension protoplasts upon co‐expression of FtMYB16‐cYFP with nYFP‐Ftimportin‐α1, while no interaction was detected between FtMYB16ΔNLS and Ftimportin‐α1 and in the negative controls (any combination of empty YFP vectors; Figure 1g). These results confirmed that FtMYB16 interacted with Ftimportin‐α1 in both plant nucleus and cytoplasm depending on the NLS motif of FtMYB16.

To investigate whether Ftimportin‐α1 is capable to transport FtMYB16 into the nucleus in plant, microscopy observations were performed in wild‐type (WT) and *mos6‐1* mutant mesophyll protoplasts. The results show that 5% of the transfected protoplasts exhibited YFP‐FtMYB16 localization in the nucleus of *mos6‐1* mutant, while 94% of that in WT protoplasts (Figure 1h). The further co‐transfection of YFP‐FtMYB16 with haemagglutinin‐tagged Ftimportin‐α1 (Ftimportin‐α1‐HA) into *mos6‐1* mesophyll protoplasts showed that a combination of Ftimportin‐α1 dramatically increased the percentage of nuclear‐localized YFP‐FtMYB16, rather than that of YFP‐FtMYB16ΔNLS (Figure 1h). Arabidopsis protoplast transactivation (APT) assays showed that Ftimportin‐α1 promoted the repression of the *FtMATE1* promoter regulated by FtMYB16, indicating the enhanced nuclear translocation of FtMYB16 mediated by Ftimportin‐α1 (Figure 1i). These results demonstrated that Ftimportin‐α1 directly mediated the translocation of FtMYB16 to the nucleus, and thereby, FtMYB16 could execute its repressive role.

To confirm that the Ftimportin‐α1 protein was involved in the regulation of FtMYB16 activity, we investigated the rutin concentration and biomass of *F. tataricum* hairy roots overexpressing and silencing *Ftimportin‐*α*1*. As shown in Figure 1j, the higher levels of rutin in the *Ftimportin‐*α*1* silencing lines and the lower levels of rutin in *Ftimportin‐*α*1* overexpressing lines compared with the EV lines were observed. In addition, the *Ftimportin‐*α*1* overexpressing lines grew slowly whereas silencing lines grew fast compared with the EV lines. These results demonstrated that Ftimportin‐α1 repressed rutin accumulation and root growth.

In summary, FtMYB16 represses the expression of not only some key enzyme genes in rutin biosynthesis, but also the *FtMATE1* gene encoding the transporter of isoquercitrin for rutin accumulation. The Ftimportin‐α1 directly regulates the import of its cargo protein FtMYB16 to nucleus, and this nucleo‐cytoplasmic trafficking signalling pathway depends on their protein interaction domain, such as NLS motif. Additionally, Ftimportin‐α1 promotes the transcriptional activity of FtMYB16 on its target genes. This study not only introduces importin‐α as a component of the regulation of rutin metabolism mediated by subgroup 4 MYB TFs, but also establishes a mode of multiple complex in the nucleo‐cytoplasmic trafficking signalling pathway in plants (Figure 1k), which may be useful for the design of artificial factors modified metabolic pathway.

## Conflict of interests

The authors declare no conflict of interests.
